# Identification of Ferroptosis-related potential biomarkers and immunocyte characteristics in Chronic Thromboembolic Pulmonary Hypertension via bioinformatics analysis

**DOI:** 10.1186/s12872-023-03511-5

**Published:** 2023-10-11

**Authors:** Jiangpeng Lin, Shuangfeng Lin, Yuzhuo Zhang, Weihua Liu

**Affiliations:** 1 Department of Cardiology, Guangzhou Institute of Cardiovascular Disease, Guangdong Key Laboratory of Vascular Diseases, The Second Affiliated Hospital, Guangzhou Medical University, Guangzhou, 510260 China; 2 The First Affiliated Hospital of Guangzhou University of Chinese Medicine, Guangzhou, 510405 China

**Keywords:** CTEPH, Ferroptosis, Biomarker, Immune cell infiltration, Machine learning

## Abstract

**Background:**

Chronic Thromboembolic Pulmonary Hypertension (CTEPH) is a form of pulmonary hypertension with a high mortality rate. A new type of iron-mediated cell death is Ferroptosis, which is characterized by the accumulation of lethal iron ions and lipid peroxidation leading to mitochondrial atrophy and increased mitochondrial membrane density. Now, there is a lack of Ferroptosis-related biomarkers (FRBs) associated with pathogenic process of CTEPH.

**Methods:**

The differentially expressed genes (DEGs) of CTEPH were obtained by GEO2R. Genes related to Ferroptosis were obtained from FerrDb database. The intersection of Ferroptosis and DEGs results in FRBs. Gene Ontology (GO) and Kyoto Encyclopedia of Genes and Genomes (KEGG) analysis were performed in Database for Annotation, Visualization and Integrated Discovery (DAVID) database. The optimal potential biomarkers for CTEPH were analyzed by least absolute shrinkage and selection operator (LASSO) and support vector machine-recursive feature elimination (SVM-RFE) machine learning. The four hub genes were verified from the Gene Expression Omnibus (GEO) dataset GSE188938. Immune infiltration was analyzed by CIBERSORT. SPSS software was used to analyze the Spearman rank correlation between FRBs identified and infiltration-related immune cells, and *p* < 0.05 was considered as statistically significant.

**Results:**

In this study, potential genetic biomarkers associated with Ferroptosis in CTEPH were investigated and explored their role in immune infiltration. In total, we identified 17 differentially expressed Ferroptosis-associated genes by GEOquery package. The key FRBs including *ARRDC3*, *HMOX1*, *BRD4*, and *YWHAE* were screened using Lasso and SVM-RFE machine learning methods.Through gene set GSE188938 verification, only upregulation of gene *ARRDC3* showed statistical difference. In addition, immune infiltration analysis using the CIBERSORT algorithm revealed the infiltration of Eosinophils and Neutrophils in CTEPH samples was less than that in the control group. And correlation analysis revealed that *ARRDC3* was positively correlated with T cells follicular helper (*r* = 0.554, *p* = 0.017) and negatively correlated with Neutrophils (r = -0.47, *p* = 0.049).

**Conclusions:**

In conclusion, *ARRDC3* upregulation with different immune cell infiltration were involved in the development of CTEPH. *ARRDC3* might a potential Ferroptosis-related biomarker for CTEPH treatment. This study provided a new insight into pathogenesis CTEPH.

**Supplementary Information:**

The online version contains supplementary material available at 10.1186/s12872-023-03511-5.

## Introduction

Chronic thromboembolic pulmonary hypertension (CTEPH) is a rare progressive pulmonary vascular disease that usually results from acute pulmonary embolism. CTEPH is characterized by persistent thromboembolic pulmonary artery obstruction and the presence of variable small vessel lesions [1]. In recent years, studies have shown that between 3 and 30 people per 1 million develop CTEPH [2]. The early symptoms of CTEPH are usually not obvious. The mortality of patients with advanced CTEPH is high, and the recent 5-year survival rate is less than 40% [3]. The pathogenesis of CTEPH is believed to be caused by multiple factors, including hypercoagulability, fibrinolysis disease, inflammation, pulmonary microvascular hyperplasia [4]. CTEPH is difficult to diagnose in early stages, and biomarkers to predict its severity and clinical outcome are still lacking. As a result, it is crucial to explore more valuable genetic biomarkers for the potential etiology and therapeutic targets of CTEPH.

Ferroptosis is characterized by mitochondrial atrophy and increased mitochondrial membrane density, the accumulation of iron and lipid reactive oxygen species and the involvement of a unique set of genes. Divalent iron ions in the unstable iron pool of the cell generate hydroxyl radicals (lipid ROS) through the Fenton reaction, causing peroxidation of polyunsaturated fatty acids. This process is accompanied by overloading or inactivation of the lipid peroxide scavenging system, and eventually the accumulation of toxic lipid metabolites leads to cell death [5]. The pulmonary circulation has the physiological basis for Ferroptosis because it is rich in blood oxygen supply, polyunsaturated fatty acids and iron ions. Although Ferroptosis was first formally proposed in tumors, more and more studies have shown that Ferroptosis is involved in the development of chronic obstructive pulmonary disease(COPD), acute lung injury, pulmonary fibrosis and other lung diseases [6]. Ferroptosis is involved in the damage of pulmonary vessels through oxidative stress and lipid peroxidation [7]. Recently, a growing number of studies have focused on discovering biomarkers of Ferroptosis at multiple levels, including morphology, biochemistry, protein, and genes [8]. However, it remains a great challenge to explore the reliable gene biomarkers and specific regulatory details associated with Ferroptosis in CTEPH.

Bioinformatics analysis is a new cross analysis combining molecular biology and information technology. The aim is to efficiently and extensively obtain differential gene expression of diseases by using gene chips, and provide signaling pathways for exploring the specific mechanisms of diseases or prediction for drug therapy [9–12]. Immune cells play an important role in the pathological process of CTEPH by participating in the release of inflammatory factors, cytotoxic effects, thrombosis and so on [13]. In the present study, we applied the CIBERSORT tool to the assessment of immune infiltration in the pathological process of CTEPH. Besides, we screened and verified Ferroptosis-related genes as CTEPH biomarkers, analyzed their role in the pulmonary vascular immune microenvironment by bioinformatics analysis. And, the pathway analysis was carried out to provide specific pathway guidance for the subsequent experimental research.

## Materials and methods

### Data download

We used the “GEOquery” package of R software (version 4.1.3) to download the CTEPH expression profile datasets GSE130391 from the Gene Expression Omnibus (GEO) (https://www.ncbi.nlm.nih.gov/geo/) database (Supplementary Material 1) [14]. Details of the GSE130391 dataset: Samples surgically extracted from CTEPH pulmonary arteries were compared to post-transplant pulmonary arteries from IPAH and failed donor pulmonary arteries from controls. The goal was to determine if altered gene expression in CTEPH associated with development of chronic thrombi. The CTEPH group (*n* = 14) and the Control group (*n* = 4) were selected for subsequent analysis.

### Differential expression analysis

We first extracted 676 differentially expressed genes (DEGs) between CTEPH and Control from GSE130391 database using GEO2R (Supplementary Material 2). DEGs with *p* < 0.05 and |log2FC|> 1 were considered significant. Genes related to Ferroptosis were obtained from FerrDb database [15]. The intersection genes of Ferroptosis and DEGs were considered to be the most important genes. Venn diagram was drawn by Venn diagram intersection (bioinformatics.psb.ugent.be/webtools/Venn/).

### Functional enrichment

GO and KEGG analysis of 17 genes were performed in Database for Annotation, Visualization and Integrated Discovery (DAVID) [16]. Metascape (http://metas.cape.org/) was used to analyse Reactome pathway enrichment and Immunologic Signatures enrichment [17].The KEGG formal permission had been obtained from Kanehisa laboratories, which copyrighted KEGG pathway database [18–20]. *p* < 0.05 was considered significant enrichment. Gene set enrichment analysis (GSEA) was performed on the gene expression matrix through Xiangtao Tool [21]. *p* < 0.05 was considered significant enrichment.

### Identification of optimal potential biomarkers for CTEPH

Glmnet package of R was applied for the LASSO algorithm. And the average misjudgment rate was compared by its tenfold cross validation in addition. SVM-RFE is a machine learning method based on support vector machine (SVM), which deletes the feature vectors generated by SVM to find the best variables. “e1071” package was used to identify the diagnostic value of these biomarkers in CTEPH [22]. The area under the curve (AUC) was used to evaluate the diagnostic ability of the best genetic biomarkers by SVM-RFE. The intersection genes of Lasso and SVM-RFE were considered as the best diagnostic gene biomarker for CTEPH. The code for Lasso and SVM-RFE machine learning algorithms has been shown in Supplementary Material 1.

### Verification of optimal potential biomarkers for CTEPH

The CTEPH-related dataset GSE188938 was obtained from the GEO database. R software was used to obtain gene expression profiles(Control, *n* = 5;CTEPH, *n* = 7). In the study of GSE188938, Professor Xu sought to identify key genes and pathways that contribute to the diagnosis and treatment of CTEPH. It provided the research direction for further understanding the molecular mechanism of CTEPH [23]. SPSS software was used for difference analysis between Control group and CTEPH group.

### Immune infiltration analysis

We uploaded GSE130391 gene expression matrix data to CIBERSORT (https://cibersort.stanford.edu/), and obtained 22 kinds of immune cell infiltration information [24]. *p* < 0.05 of the samples were identified significantly.“corrplot” package and “ggplot2” package were used to draw a correlation heatmap and boxplot. The proportion of 22 types of infiltrating immune cell types in each tissue from the GSE130391 dataset were showed in Supplementary Material 5.

### Correlation analysis between diagnostic biomarkers and infiltration-related immune cells

SPSS software was used to analyze the Spearman rank correlation between FRBs identified and the level of related immune cells. *P* < 0.05 was considered statistically significant [25]. We also uesed "ggplot2" package in R software to diplay the correlation.

### Statistical analysis

The data were analyzed by one-way Analysis of Variance(ANOVA) to determine differences between the Control group and the CTEPH group. For all statistical tests, *p* < 0.05 was considered as statistically significant.

## Results

### Identification of Ferroptosis related genes and DEGs in CTEPH

**Fig. 1:**
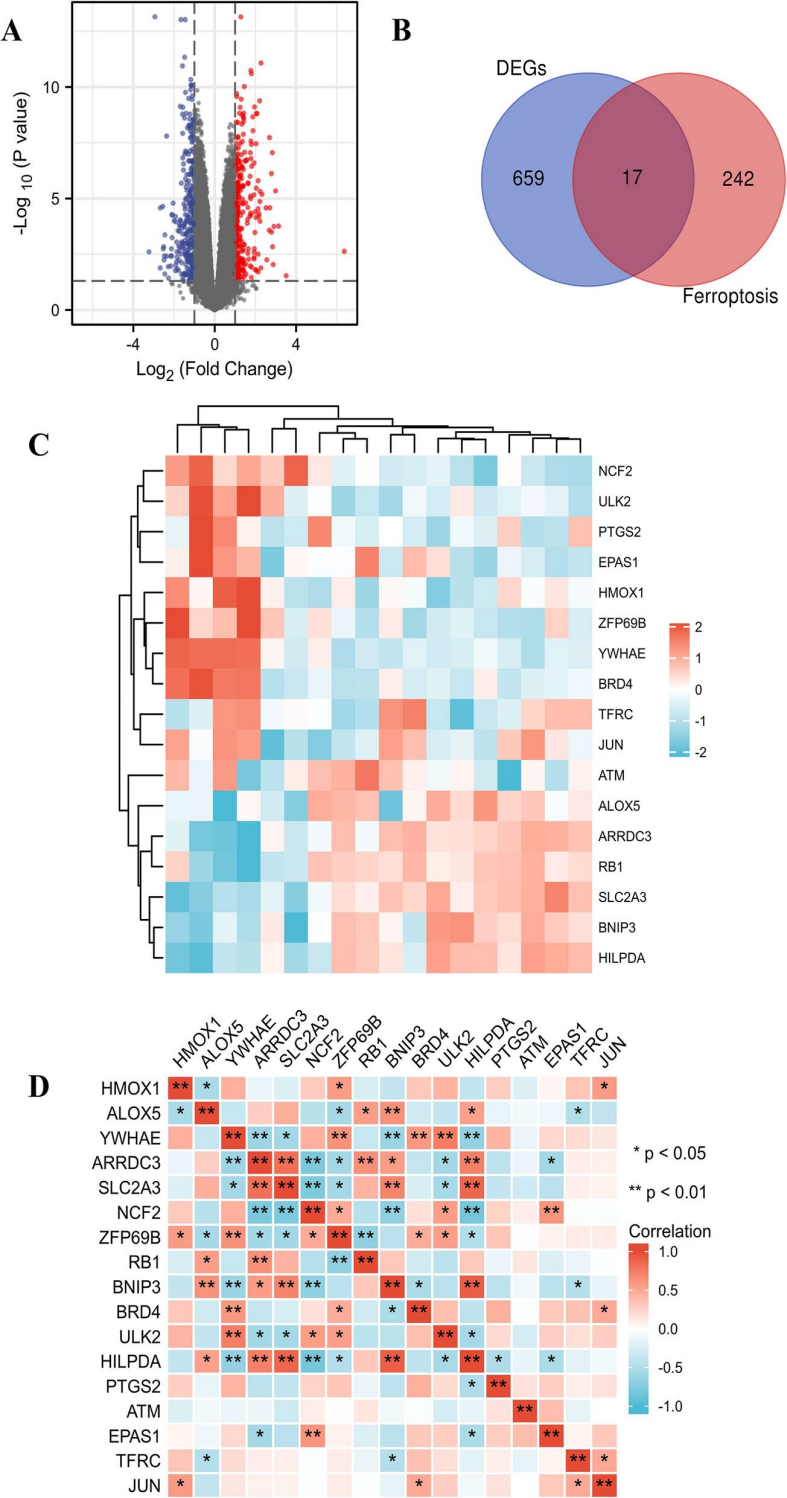
Identification of Ferroptosis  related genes and DEGs in CTEPH. **A** PCA diagram of the DEGs expression with *p* < 0.05 and |log2FC|> 1. Red represents genes up-regulation. Blue represents genes down-regulation. **B** Venn diagram of Ferroptosis and DEGs. **C** The clustering heatmap of 17 Ferroptosis related intersection genes. **D** The correlation between 17 intersection genes. ^*^p < 0.05, ^**^p < 0.01

There were 676 differentially expressed genes (DEGs) between CTEPH and Control from GSE130391 database. PCA diagram showed the expression of these genes (Fig. 1A). 259 genes related to Ferroptosis were obtained from FerrDb database (Supplementary Material 3). There were 17 intersection genes of Ferroptosis and DEGs (Fig. 1B, Table 1). The clustering heatmap showed the expression pattern of 17 Ferroptosis related biomarkers (FRBs) among samples (Fig. 1C). The correlation between these 17 genes were shown in Fig. 1D.

**Table 1:** 17 Ferroptosis-related biomarkers of Ferroptosis and DEGs

Gene	p- Value	Expressing trend	Gene ID	The link of gene information
*HMOX1*	0.0000983	down	3162	https://www.ncbi.nlm.nih.gov/gene/3162
*EPAS1*	0.0034	down	2034	https://www.ncbi.nlm.nih.gov/gene/2034
*YWHAE*	3.25E-10	down	7532	https://www.ncbi.nlm.nih.gov/gene/7531
*JUN*	0.0012	down	3725	https://www.ncbi.nlm.nih.gov/gene/3725
*ULK2*	0.00000456	down	9706	https://www.ncbi.nlm.nih.gov/gene/9706
*BRD4*	1.32E-10	down	23,476	https://www.ncbi.nlm.nih.gov/gene/23476
*NCF2*	0.00372	down	4688	https://www.ncbi.nlm.nih.gov/gene/4688
*ZFP69B*	0.0000634	down	65,243	https://www.ncbi.nlm.nih.gov/gene/65243
*PTGS2*	0.00509	down	5743	https://www.ncbi.nlm.nih.gov/gene/5743
*RB1*	0.000413	up	5925	https://www.ncbi.nlm.nih.gov/gene/5925
*ATM*	0.00000589	up	472	https://www.ncbi.nlm.nih.gov/gene/472
*BNIP3*	0.000212	up	664	https://www.ncbi.nlm.nih.gov/gene/664
*TFRC*	3.52E-10	up	7037	https://www.ncbi.nlm.nih.gov/gene/7037
*ALOX5*	0.03	up	240	https://www.ncbi.nlm.nih.gov/gene/240
*ARRDC3*	0.00000483	up	57,561	https://www.ncbi.nlm.nih.gov/gene/57561
*SLC2A3*	0.0000263	up	6515	https://www.ncbi.nlm.nih.gov/gene/6515
*HILPDA*	0.000173	up	29,923	https://www.ncbi.nlm.nih.gov/gene/29923

### Functional enrichment of 17 Ferroptosis-related DEGs

**Fig. 2:**
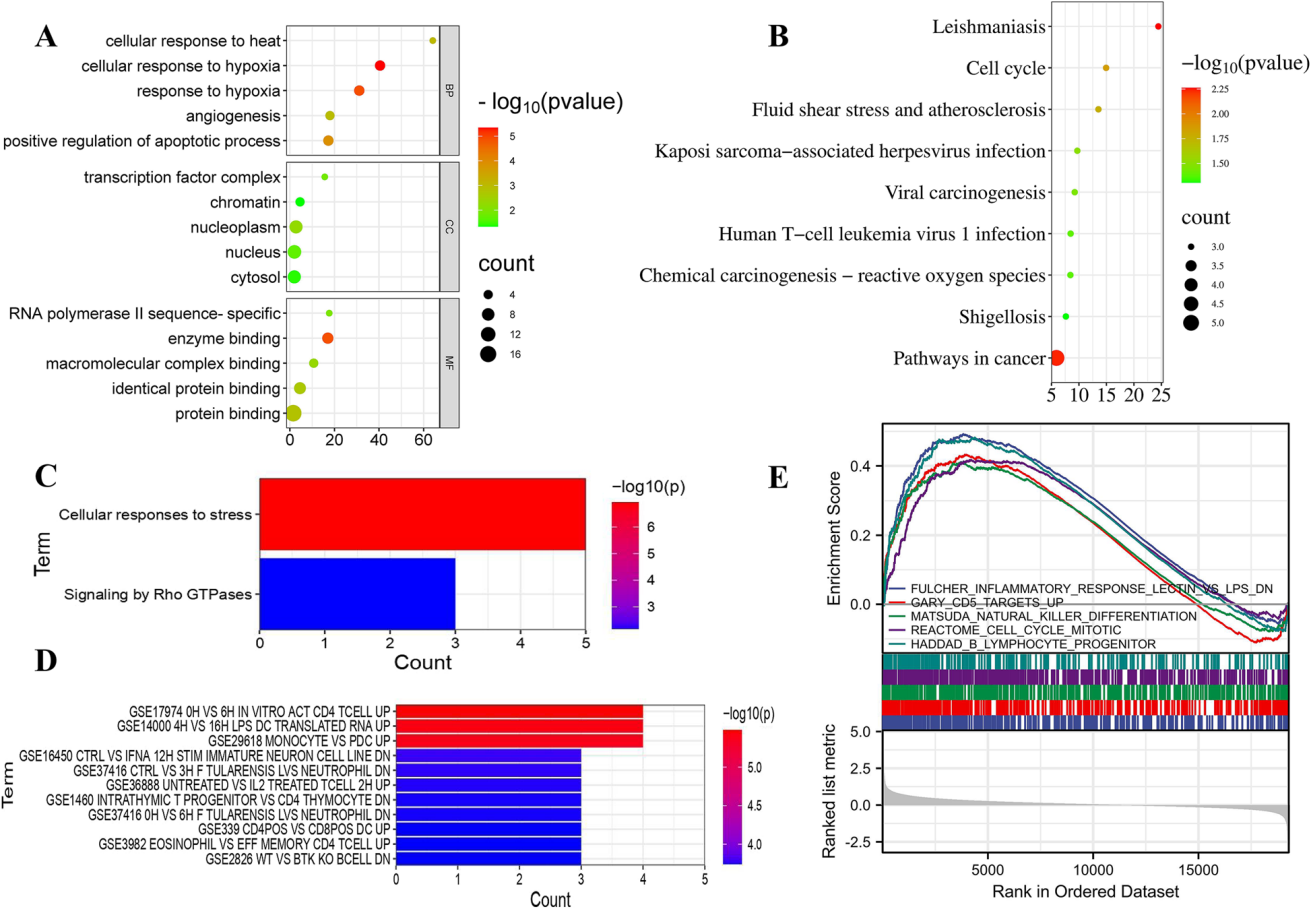
Functional enrichment and GSEA enrichment analysis of 17 Ferroptosis-related genes. **A** GO enrichment analysis of 17 intersection genes. Biology Process (BP), Molecular Function (MF), Cellular Componet (CC). **B** KEGG enrichment analysis.The KEGG formal permission had been obtained from Kanehisa laboratories, which copyrighted KEGG pathway database. Y-axis indicates GO or KEGG entries and X-axis indicates the number of genes enriched under the entry, the redder the color, the smaller the value of p value, which means more significant enrichment.The bubble diagram X-axis indicates the ratio of genes, the larger the circle, the more genes are present in each pathway. **C** Reactome pathway enrichment analysis. **D** Immunologic Signatures enrichment analysis. **E** GSEA enrichment analysis. The top part is the line chart of the top 5 gene Enrichment scores. The horizontal axis is each gene that corresponds to this gene set, and the vertical axis is the corresponding Running Enrichment Score. The peak is the Enrichemnt score of the gene set, and the genes before the peak are the core genes under the gene set. The middle part is hit, and the genes under this gene set are marked with lines. The bottom part is the distribution of rank values for all genes

GO enrichment analysis indicated that the above 17 Ferroptosis related biomarkers were significantly related to regulation of inflammatory response, Cell growth cycle, cell death, iron ion homeostasis, mitophagy and so on (Fig. 2A). KEGG analysis showed 17 FRBs significantly enriched in Leishmaniasis, Pathways in cancer, Cell cycle, etc. (Fig. 2B). Reactome pathway analysis indicated that Cellular responses to stress and Signaling by Rho GTPases were enriched (Fig. 2C). Surprisingly, the 17 FRBs were also obviously enriched in many immune-related signatures (Fig. 2D). It was showed the top five GSEA enrichment gene sets involved in inflammation, CD5 cells, natural killer cells and B lymphocyte in Fig. 2E. Collectively, These evidences indicated that 17 intersection genes mentioned may play an important role in the pathogenesis of CTEPH by participating in the regulation of immune cells and cytokines.

### Screening and verification of potential biomarkers for CTEPH

**Fig. 3:**
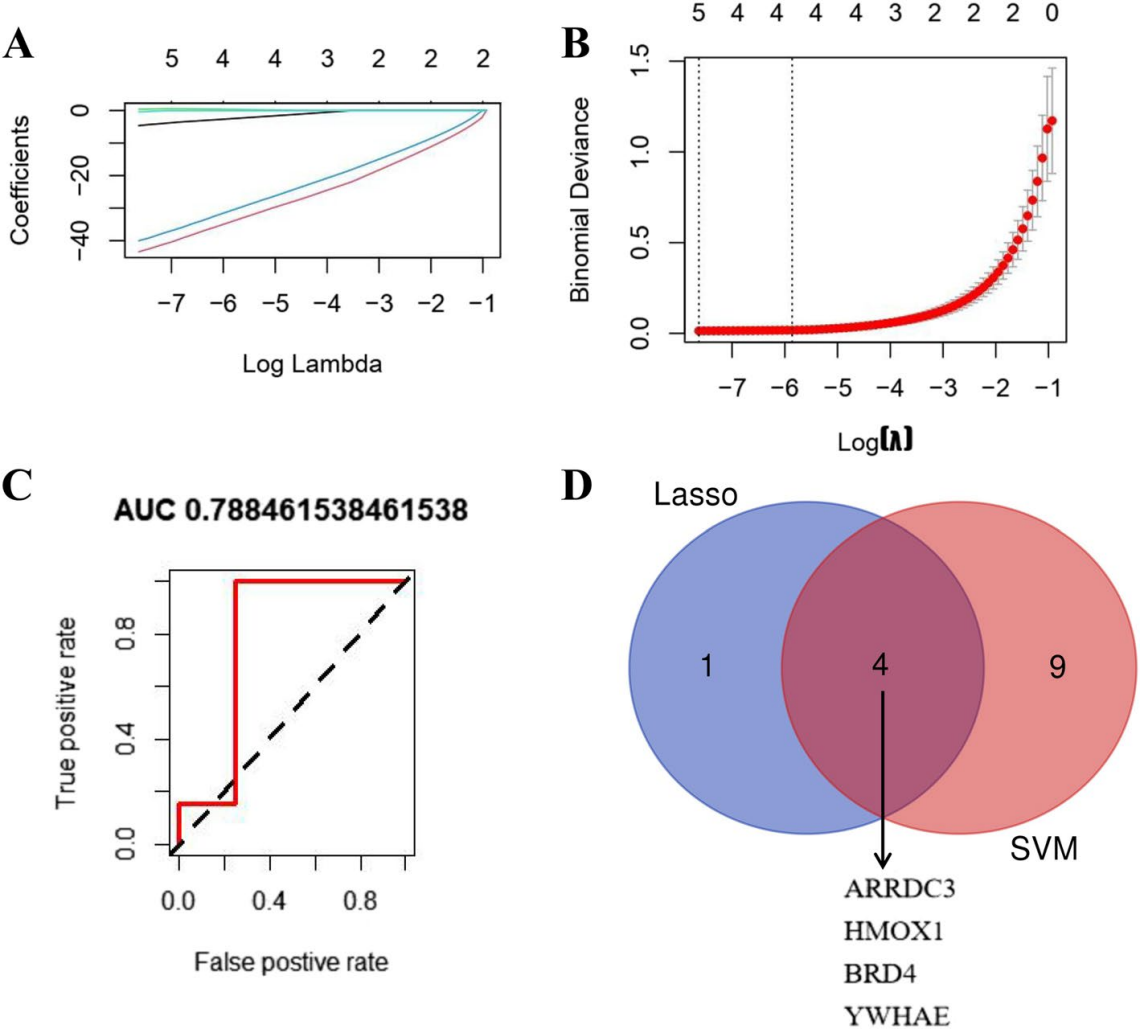
Screening and verification of potential biomarkers for CTEPH. **A** LASSO logistic regression algorithm, with penalty parameter tuning conducted by 10- fold cross-validation. Five different colored lines represent five important diagnostic markers that LASSO has identified. **B** The misclassification error in the jackknife rates analysis. The number of genes (*n* = 5) corresponding to the lowest point of the curve is the most suitable number for CTEPH Ferroptosis-related potential biomarkers using Lasso algorithm. **C** The diagnostic efficiency of SVM-RFE. The closer the AUC is to 1, the better the result of gene selection. **D** The intersection potential biomarkers of the Lasso and SVM-RFE. LASSO, least absolute shrinkage and selection operator; SVM-RFE, support vector machine recursive feature elimination

We screened out 5 genes from 17 FRBs as the most suitable potential biomarkers for CTEPH by using LASSO algorithm (Fig. 3A-B). 13 potential biomarkers were selected from DEGs by SVM-RFE algorithm. AUC = 0.788 indicated that SVM-RFE had a good gene selection effect (Fig. 3C). The intersection potential biomarkers obtained by the two algorithms were considered the most accurate potential biomarkers, including *ARRDC3*, *HMOX1*, *BRD4* and *YWHAE *(Fig. 3D).

### Verification of four hub genes from the GEO dataset GSE188938

**Fig. 4:**
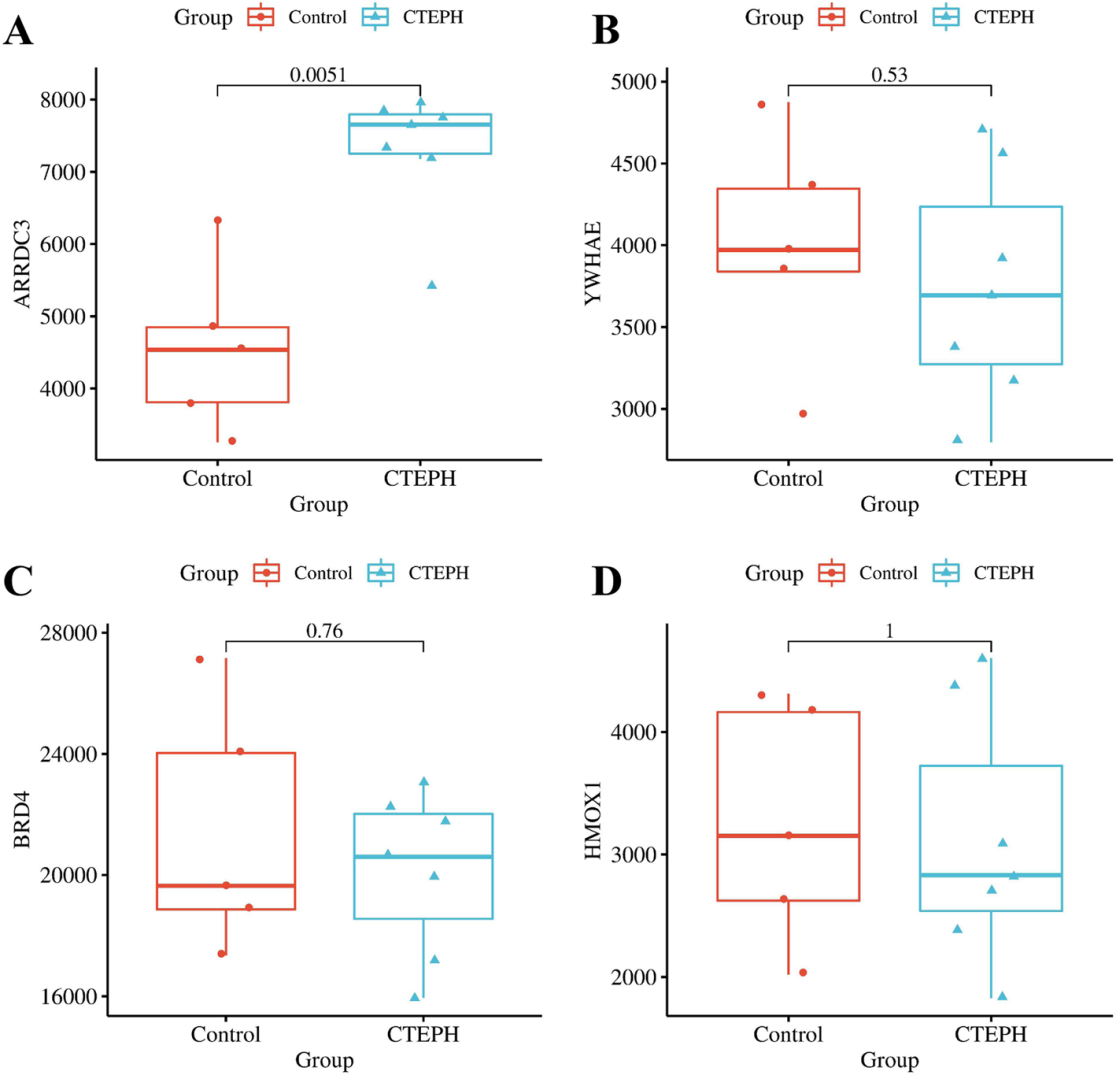
Verification of four hub genes from the GEO dataset GSE188938. *p* < 0.05 indicates that there is a statistical difference between the CTEPH group and the Control group. Red represents the Control group. Blue represents the CTEPH group

In the GEO dataset GSE188938, *ARRDC3* was significantly upregulated in CTEPH group compared with control group (*p* = 0.0051, Fig. 4A). However, we found that the gene expression of *HMOX1*, *BRD4* and *YWHAE* were not statistically significant (Fig. 4B-D).These results indicated that *ARRDC3* may be the most important hub gene of CTEPH and a potential therapeutic target. The expression of *ARRDC3*, *HMOX1*, *BRD4* and *YWHAE* in GSE188938 were showed in Supplementary Material 4.

### Immune infiltration analysis

**Fig. 5:**
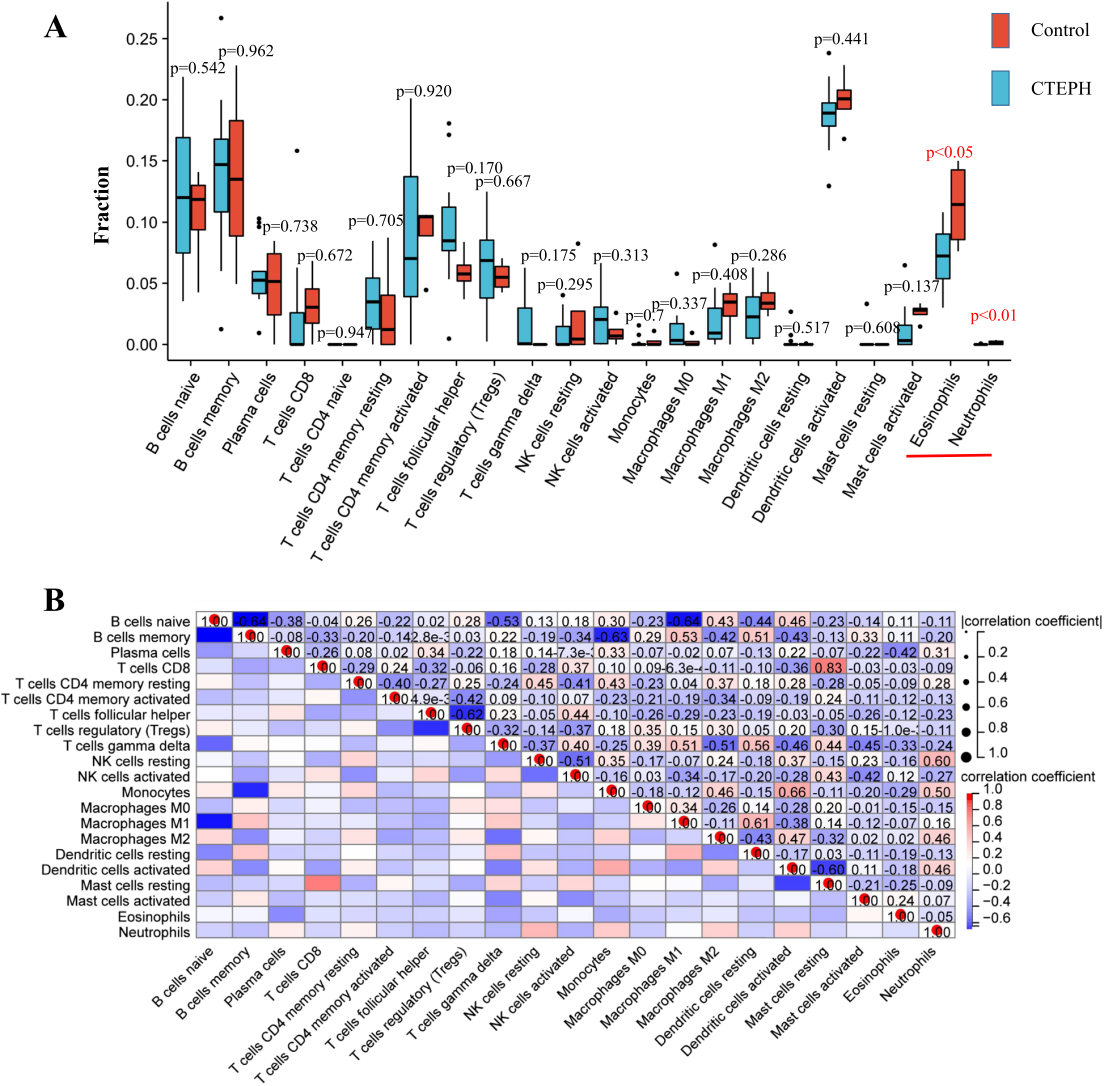
Immune infiltration analysis. **A** The boxplot of the immune cell. Red represents the Control group. Blue represents the CTEPH group. Red represents the Control group. Blue represents the CTEPH group. **B** Correlation heatmap of Immune infiltration analysis.Red means positive correlation. Blue represents the negative correlation

The boxplot of the immune cell infiltration difference showed a reduction in Eosinophils and Neutrophils at CTEPH compared with Control samples (Fig. 5A). In correlation heatmap, Pearson correlation analysis of the 22 types of immune cells reveals that Eosinophils is negatively correlated with Neutrophils (Fig. 5B). As a result, Eosinophils and Neutrophils may be involved in the pathological regulation of CTEPH.

### Correlation analysis between *ARRDC3* and infiltration-related immune cells

**Fig. 6:**
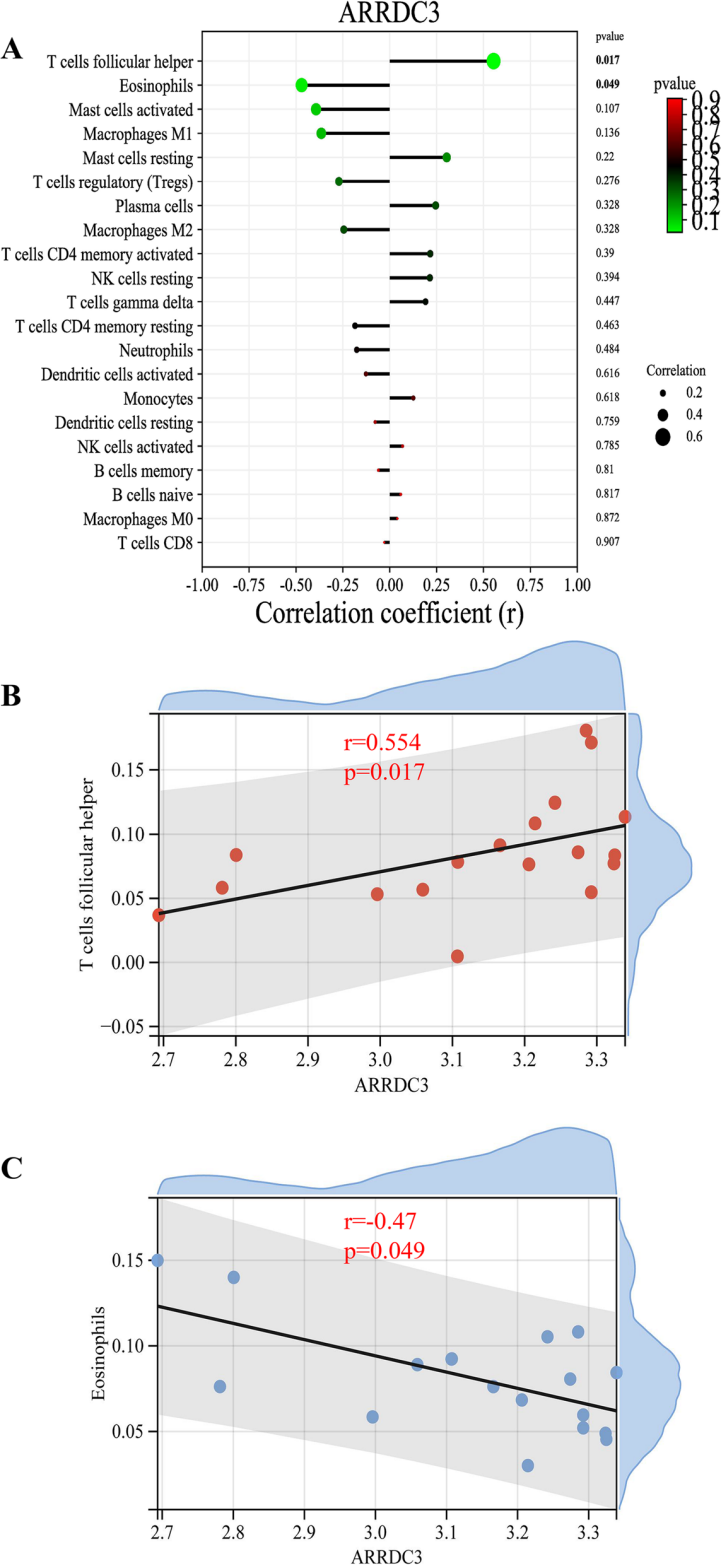
Correlation analysis between *ARRDC3* and infiltration-related immune cells. **A** The correlation between *ARRDC3* and Infiltrating Immune Cells. **B** Scatter diagram indicates the correlation between *ARRDC3* expression and T cells follicular helper. The expression of *ARRDC3* is positively correlated with T cells follicular helper (*r* = 0.554, *p* = 0.017). **C** Scatter diagram indicates the correlation between *ARRDC3* expression and Neutrophils. The expression of *ARRDC3* is negatively correlated with Neutrophils (*r* = -0.47, *p* = 0.049)

Correlation analysis revealed that *ARRDC3* was positively correlated with T cells follicular helper (*r* = 0.554, *p* = 0.017, Fig. 6A-B). *ARRDC3* was negatively correlated with Neutrophils (*r* = -0.47, *p* = 0.049, Fig. 6C, Supplementary Material 5).

## Discussion

Despite the advancements in CTEPH treatment during the past decades, pulmonary endarterectomy (PEA) treatment has not been completely satisfactory and there are still some patients with recurrent attacks. With the development of sequencing technology, it is increasingly expected to uncover the biological effects of genes related to CTEPH. Ferroptosis is believed to involve in the development of vascular inflammation and thrombosis. However, few studies have focused on the genes and underlying regulatory details associated with iron deficiency and the immune infiltration landscape, which have profound implications for patients with CTEPH. In this study, a total of 17 differentially expressed genes related to Ferroptosis in CTEPH patients were identified in the metadata cohort. Among them, four genes including *ARRDC3*, *HMOX1*, *BRD4*, and *YWHAE* are considered as key genes for Ferroptosis in CTEPH development by the integration of LASSO and SVM-RFE machine learning methods. Only the upregulation of *ARRDC3* was found to have a statistical difference through gene set GSE188938 verification. In addition, analysis of immune infiltration using the CIBERSORT algorithm revealed less infiltration of Eosinophils and Neutrophils in CTEPH samples than in controls. *ARRDC3* was found to be positively correlated with T cells follicular helper and negatively correlated with Neutrophils according to the correlation analysis.

Firstly, We downloaded the CTEPH expression profile dataset GSE130391 from the GEO database and identified a total of 676 DEGs on CTEPH. There were 17 intersection genes of Ferroptosis and DEGs, indicating that Ferroptosis is likely to be involved in the pathogenesis of CTEPH through these 17 genes. GO enrichment analysis revealed that 17 intersection genes were mainly associated with regulation of inflammatory response, regulation of cell cycle and cell death. In the results of KEGG enrichment analysis, there were significantly enriched in pathways of cancer, cell cycle, chemical carcinogenesis-reactive oxygen species. Evidences have confirmed that immune response participated in CTEPH development [26] and chronic inflammatory diseases including inflammatory bowel disease, osteomyelitis and anticardiolipin antibody syndrome and chronic venous ulcers would increase the risk of CTEPH [27]. CTEPH pulmonary vascular remodeling could be attributed partly to the abnormal gene regulation of cell cycle and cell death via apoptosis and Ferroptosis in pulmonary artery endothelial cells and smooth muscle cells [5, 28]. These studies mentioned above were consistent with our GO and KEGG results. In the present study, Immunologic Signatures enrichment analysis also indicated that Ferroptosis-related genes were involved in CTEPH pathogenesis by regulating autophagy, immune cells, cytokines and multiple kinases. In addition, the main gene sets enriched in GSEA analysis were NFLAMMATORY RESPONSE, CD5 TARGETS, NATURAL KILLER DIFFERENTIATION, CELL CYCLE MITOTIC, HADDAD B LYMPHOCYTE PROGENITOR. All above results suggested that Ferroptosis and immune inflammatory response might play important roles in CTEPH progression.

Next, four genes including *ARRDC3*, *HMOX1*, *BRD4*, and *YWHAE* were identified as key hub genes in CTEPH combined with Ferroptosis by LASSO and SVM-RFE algorithms which are mainly used to filter the most characteristic variables and construct the best classification models [26]. *ARRDC3* is associated with inflammation, cellular invasion, etc., which promotes H. pylori-associated gastritis by regulating protease-activated receptor 1 and inhibits invasive metastasis of breast cancer cells by regulating G protein-coupled receptor lysosomes [29]. *HMOX1* could regulate the proliferation and apoptosis of human cardiomyocytes and play an anti-oxidative stress role in apoptosis induced by high glucose [30, 31]. *BRD4* inhibition prevented diabetic cardiomyopathy induced by high-fat diet through activating mediated mitochondrial autophagy [32]. *YWHAE* promoted proliferation and metastasis of breast cancer cells, and regulated proliferation and cell cycle protein D1 through RAF/MAPK and Hippo pathways [33]. The above studies indicated that *ARRDC3*, *HMOX1*, *BRD4*, and *YWHAE* play a role in promoting inflammation, cell proliferation and metastasis, and abnormal cell apoptosis. The pathogenesis of CTEPH is closely related to chronic inflammation, abnormal cell proliferation and abnormal cell cycle regulation [34]. In this study, our results indicated that *ARRDC3*, *HMOX1*, *BRD4*, and *YWHAE* were involved in CTEPH progress possibly through regulating inflammatory, vascular cell proliferation, smooth muscle cell invasion and apoptosis. Through gene set GSE188938 verification, only *ARRDC3* upregulation showed statistical difference suggesting *ARRDC3* might be the most important hub gene of CTEPH and a potential therapeutic target for CTEPH.

Additionally, we screened the proportion of 22 immune cells in CTEPH using CIBERSORT analysis, and found that there were significant differences in the cells infiltration of Eosinophils and Neutrophils compared with the control sample. We also analyzed the correlation between *ARRDC3* and immune cells infiltration. *ARRDC3* was positively correlated with T cells follicular helper and negatively correlated with Neutrophils. Eosinophils and Neutrophils were believed to be involved in pulmonary artery atherosclerosis and thrombotic lesions leaded to CTEPH [35, 36]. Ferroptosis-inducing agents induced Ferroptosis-like cell death of Eosinophils [37]. Neutrophil extracellular traps mediated m6A modification and regulated sepsis-associated acute lung injury by activating Ferroptosis in alveolar epithelial cells [38]. These studies were similar with our results that both Eosinophils and Neutrophils were involved in Ferroptosis processes of CTEPH. Our study found that Ferroptosis-related hub gene *ARRDC3* was related to immune cell infiltration including T cells follicular helper and Neutrophils, and further experiments are necessary to verify its exact effect and underlying regulatory mechanism.

The results of bioinformatics analysis provided a new clue and viewpoint for our further research on CTEPH. Undeniably, there were some limitations in the current study, such as the sample size of the selected database, heterogeneity of the clinical parameters of CTEPH patients and lack of grading of disease severity. To minimize sample heterogeneity, it is necessary to include more samples and improve clinical data. Further experimental verification are warranted to confirm the effects and functional correlation of the identified genes and pathways, and provide more accurate characterization of immune cell populations in CTEPH in vivo and vitro studies.

In conclusion, *ARRDC3* upregulation with different immune cell infiltration were involved in the development of CTEPH and *ARRDC3* might a potential and promising Ferroptosis-related biomarker for CTEPH treatment. Our study might provide new insights into the prevention, monitoring and potential therapeutic intervention for CTEPH.

### Supplementary Information

**Additional file 1:** **Supplementary Material 1.** Data of GSE130391.

**Additional file 2:** **Supplementary Material 2.** 676 DEGs between CTEPH and Control group.

**Additional file 3:** **Supplementary Material 3.** 259 Ferroptosis-related genes.

**Additional file 4:** **Supplementary Material 4.** The expression of ARRDC3, HMOX1, BRD4 and YWHAE in GSE188938.

**Additional file 5:** **Supplementary Material 5.** The expression of 22 immune cells in the sample and Correlation analysis.

## Data Availability

The data used to support the findings of this study are available from Supplementary Material.

## Abbreviations

CTEPH: Chronic thromboembolic pulmonary hypertension
FRBs: Ferroptosis related biomarkers
DEGs: Differentially expressed genes
GO: Gene ontology
KEGG: Kyoto encyclopedia of genes and genomes
GSEA: Gene set enrichment analysis
GEO: Gene expression omnibus
LASSO: Least absolute shrinkage and selection operator
SVM-RFE: Support vector machine-recursive feature elimination
COPD: Chronic obstructive pulmonary disease
DAVID: Database for annotation, visualization and integrated discovery
